# The Complementary and Alternative Medicine for Antithrombosis

**DOI:** 10.1155/2015/791503

**Published:** 2015-05-21

**Authors:** Feng-Qing Yang, Chun-Su Yuan, Swee Ngin Tan, Jian-Li Gao

**Affiliations:** ^1^School of Chemistry and Chemical Engineering, Chongqing University, No. 174, Shazhengjie Street, Shapingba, Chongqing 400030, China; ^2^Tang Center for Herbal Medicine Research, The Pritzker School of Medicine, University of Chicago, 5841 South Maryland Avenue, MC 4028, Chicago, IL 60637, USA; ^3^Natural Sciences and Science Education Academic Group, Nanyang Technological University, 1 Nanyang Walk, Singapore 637616; ^4^Zhejiang Chinese Medical University, No. 548 Binwen Road, Binjiang, Hangzhou, Zhejiang 310053, China

Blood stasis syndrome (BSS) or blood stagnation is often understood in terms of hematological disorders such as thrombosis, congestion, hemorrhage, and local ischemia (microclots). In reality, BSS or thrombosis is considered to be closely related to senile diseases such as atherosclerosis, ischemic heart disease, and stroke, as well as rheumatoid arthritis, hyperuricemia, and various inflammatory conditions. Therefore, more studies have been undertaken to focus on preventing thrombosis for the treatment of those thrombotic diseases. It is well known that the complementary and alternative medicines (CAMs) such as traditional Chinese medicines (TCMs) have a long history for treating thrombosis or BBS. This special issue is focused on the evidence-based research of the CAMs in antithrombotic therapies and therefore brings more attention from readers and researchers on complementary and alternative therapies for thrombosis.

The formation of thrombosis is highly complex. Understanding the molecular functions of thrombosis network is helpful for predicting potential targets for the treatment of thrombosis. X. Kong et al. retrieved pathways and reaction information related to thrombosis from the Reactome database (a peer-reviewed pathway database), then split the complex into separate single proteins, reconnected splitting proteins and detected the relations between the proteins with reacting directions, and finally mapped the integrative and comprehensive molecular network of thrombosis by combining platelet signaling, the coagulation cascade, and natural clot dissolution systems. Moreover, the topological characteristics of the network including the centralities of nodes, network modules, and network robustness were analyzed. However, further pharmaceutical experiments are necessary for eventual validation of network results.

In order to reveal the antiatherosclerotic molecular mechanisms of Astragaloside IV (AsIV) from Huangqi (*Astragalus membranaceus*), H. Qin et al. investigated the effects of AsIV on blood lipids, CD40-CD40L signal system, and SDF-1/CXCR4 biological axis in high-fat diet ApoE^−/−^ mice. Their results proved that AsIV can alleviate the extent of atherosclerosis in aorta of ApoE^−/−^ mice. And AsIV can significantly downregulate PAC-1, CD40L, and CXCR4 expression on platelet surface in blood, as well as mRNA and protein level of SDF-1, CXCR4 in thoracic aorta of the model mice. On the other hand, AsIV could downregulate TG, TC, and LDL-C levels and upregulate HDL-C levels in model mice's blood. Therefore, they made a conclusion that the protective effects of AsIV in atherosclerotic injury may be related to regulating blood lipids, CD40-CD40L system, and SDF-1/CXCR4 biological axis.

Safflower Yellow Injection (SYI) has been reported for the treatment of acute cerebral infarction. L.-J. Li et al. investigated the effects of SYI on the patients with acute cerebral infarction. Their results indicated that the scores of National Institute of Health Stroke Scale significantly decreased in the SYI treated group (after treatment for 7 and 14 d) as compared to the control group (placebo injection). Furthermore, the hemorheological index including index of red blood cell (RBC) deformation and aggregation were significantly different before and after treating with SYI. Meanwhile, the value of prothrombin time (PT) increased, and the value of fibrinogen (FIB) decreased in the SYI treated group. In addition, the serum levels of TNF-*α*, IL-1*β*, and IL-6 in the SYI group decreased as compared to the control group. The data suggests that SYI therapy may be beneficial for the patients with acute cerebral infarction.

In another study, S. Mahmud et al. investigated the antithrombotic properties (clot lysis effects) of cold methanol extracts of five Bangladeshi plants* in vitro*. Seven secondary metabolites including alkaloids, flavonoids, steroids, tannins, saponins, phlobatannins, and cardiac glycosides were identified in the experimental extracts by phytochemical screening. Among those herbal extracts,* Leea macrophylla* showed the most prominent antithrombotic effects, while* Andrographis paniculata* possessed moderate activity. Moreover,* in silico* docking simulation showed that glycosides molecule fit the best to the activation of tissue plasminogen activator, which suggested that glycosides of these plants are to be the major components contributing to the observed thrombolytic effects.

There is considerable interest in the role of natural products and their bioactive components in the prevention and treatment of thrombosis related disorders. In the review paper by C. Chen et al., the mechanisms of thrombus formation were briefly described on three aspects including coagulation system, platelet activation and aggregation, and change of blood flow conditions. And the natural products for antithrombosis by anticoagulation (inhibition of tissue factors and the coagulation pathways), antiplatelet aggregation (inhibition of platelet membrane receptors functions and granules secretion, impacting on nucleotide and arachidonic acid systems), and fibrinolysis were summarized, respectively.

It is believed that advances in the understanding of both the mechanisms of thrombus formation and the antithrombotic functions of CAMs will provide new insights to promote human health by preventing or treating thrombotic disorders.

*Feng-Qing Yang* *Feng-Qing Yang* *Chun-Su Yuan* *Chun-Su Yuan* *Swee Ngin Tan* *Swee Ngin Tan* *Jian-Li Gao* *Jian-Li Gao*

